# Legalizing Dissection

**Published:** 1873-01-15

**Authors:** 


### MEDICAL EXAMINER.

*A Semi-Monthly Journal of Medical Sciences.*

EDITED BY

N. S. DAVIS, M. D., AND F. H. DAVIS, M. D.

Chicago, January 15th, 1873.

### EDITORIAL.

### LEGALIZING DISSECTION.

The season for the assembling of the Legislature of this and other States, reminds us of the fact that we are yet without legal provision for the study of human anatomy, or even for the procurement of sufficient subjects on which to demonstrate the more important operations of surgery. That the necessities and sufferings of every community imperatively require the services of physicians and surgeons of intelligence and skill, is a proposition too plain to require either illustration or argument. That a thorough personal study of all the tissues and organs in the human body is absolutely essential to constitute either, is also a self-evident truth. That such study can be carried on efficiently in no other way than by actual dissection, is equally obvious to every intelligent citizen. There is not a member of the Legislature, nor a man or woman in the community, who would be willing to employ a surgeon to tie a bleeding vessel or set a broken limb for themselves, who had never actually seen the vessel he proposed to tie, or examined by actual dissection the joints to be replaced.

And yet, even to the present time, the laws of this State not only make no provision by which the medical colleges in the State can get the *materiel* necessary for the professors and students to dissect, but they throw every possible obstacle in the way, and exhibit much greater care for the body of a dead pauper or criminal than for the health and lives of the living citizens. It is fully acknowledged by legislators and all intelligent citizens, that the study of anatomy and surgery by dissections is absolutely necessary to fit any student for the practice of medicine and surgery. Then why not at once so modify the laws of the State as to make adequate and proper provision for supplying the medical schools and teachers with the *subjects* they need, instead of compelling them to violate law and encounter personal danger, or submit to burdensome pecuniary sacrifices in inducing others to do so? Suitable and proper laws legalizing dissections and providing for the needed supply of *subjects*, have existed for many years in Massachusetts and New York, and the same have been more recently passed in Michigan, Iowa and several other States. This has effectually stopped all attempts to invade the cemeteries in those States, and enabled their medical schools to obtain an abundant and cheap supply of *materiel* for legitimate use. This gives the medical schools in those States a decided and important advantage over those in States where no such laws exist. Let the members of the profession throughout this State immediately communicate with the members of the Legislature in their respective districts, and urge upon them the necessity of passing a law similar to those enacted in other States on this subject, early during the present legislative session.

